# Co-action provides rational basis for the evolutionary success of Pavlovian strategies

**DOI:** 10.1038/srep30831

**Published:** 2016-08-01

**Authors:** V. Sasidevan, Sitabhra Sinha

**Affiliations:** 1 The Institute of Mathematical Sciences, CIT Campus, Taramani, Chennai 600113, India

## Abstract

Strategies incorporating direct reciprocity, e.g., Tit-for-Tat and Pavlov, have been shown to be successful for playing the Iterated Prisoners Dilemma (IPD), a paradigmatic problem for studying the evolution of cooperation among non-kin individuals. However it is an open question whether such reciprocal strategies can emerge as the rational outcome of repeated interactions between selfish agents. Here we show that adopting a co-action perspective, which takes into account the symmetry between agents - a relevant consideration in biological and social contexts - naturally leads to such a strategy. For a 2-player IPD, we show that the co-action solution corresponds to the Pavlov strategy, thereby providing a rational basis for it. For an IPD involving many players, an instance of the Public Goods game where cooperation is generally considered to be harder to achieve, we show that the cooperators always outnumber defectors in the co-action equilibrium. This can be seen as a generalization of Pavlov to contests involving many players. In general, repeated interactions allow rational agents to become aware of the inherent symmetry of their situation, enabling them to achieve robust cooperation through co-action strategies - which, in the case of IPD, is a reciprocal Pavlovian one.

Understanding how cooperation can emerge in a society, each of whose individual members are seeking to maximize their personal well-being, is one of the fundamental problems in evolutionary biology and social sciences^1^,^2^,^3^. The ever present temptation to not cooperate (thereby avoiding the cost associated with such an action) while enjoying the benefits of the cooperative acts of others appears to make cooperation unstable even if it arises by chance. Yet cooperation is seen to occur widely in nature, ranging from communities of micro-organisms^4^,^5^, cellular aggregates^6^ and synthetic ecologies^7^ to primate societies^8^. The fragility of cooperation among unrelated individuals (i.e., non-kin) has been conceptually formalized in terms of the Prisoner’s Dilemma (PD) game^9^,^10^ which demonstrates how the pursuit of maximal individual benefit could lead to a collective outcome that is disastrous for all. Extensive investigation of this model system has revealed that in the Iterated PD (IPD), where repeated interactions are allowed between the same pair of individuals, successful strategies typically use information about previous interactions to choose the current action^11^. In other words, these strategies embody the phenomenon of direct reciprocity that can lead to the evolution and maintenance of cooperation^12^. Empirical evidence from experiments with human and animal subjects have been put forward in support of this notion that cooperative behavior towards other individuals is conditioned on the past actions of agents^13^,^14^,^15^,^16^,^17^,^18^,^19^,^20^,^21^.

One of the most well-known strategies incorporating direct reciprocity is tit-for-tat (TFT) where each agent initially cooperates and then imitates the preceding action of its opponent in all subsequent rounds^1^. This deceptively simple strategy has been shown to be successful in computer tournaments where different strategies compete in playing IPD with each other. However, it is known that TFT is vulnerable to noise arising out of misunderstanding of intent, errors in perception and mistakes in implementing their actions by the players - situations that arise in most natural situations. In such noisy environments, robust cooperation can result from other strategies such as generous tit-for-tat (GTFT) which forgives a defection with a small probability, contrite tit-for-tat (CTFT) that follows an unintentional defection with unconditional cooperation and Pavlov which repeats its prior move if it has been rewarded with a sufficiently high payoff but changes its behavior on being punished with a low payoff.

While these ingenious behavioral rules have been highly effective in tournaments where they compete in playing IPD against a variety of other strategies, it is unclear how one would have arrived at them as the solution to a rational decision problem. An *ab initio* derivation of such a strategy incorporating direct reciprocity as a rational solution to IPD will not only provide a theoretical breakthrough but may unveil new tools for addressing different strategic interaction problems. In this paper we show that considering the symmetry between the players - a relevant consideration in biological and social contexts - by using a co-action perspective^22^, allows us to obtain a rational solution for IPD, which we show to be same as Pavlov. Even when uncertainty about the actions of other agents and errors in implementing strategies causes agents to defect occasionally, the information about earlier moves can help the agents in inferring the underlying symmetry of the situation and thereby restore cooperation, which provides a fresh perspective on Pavlovian strategies. More importantly, we generalize this approach to the case of IPD with many players to address the question of cooperation in Public Goods dilemmas where cooperation is generally considered to be harder to achieve^23^. Surprisingly, we find that the cooperators always outnumber the defectors in the co-action equilibrium of the *N*-person IPD - which we propose as the extension of Pavlov to multi-player games. Our results show that co-action provides a general framework to understand why it is rational to cooperate even when it is lucrative to act otherwise.

In the next section we first present a summary of the co-action solution concept for single-stage (or one-shot) strategic interactions^22^ that is the appropriate framework for analyzing games under conditions of complete symmetry. As the knowledge of such symmetry may itself become apparent to agents through repeated interactions, we next extend the co-action principle to an iterative setting. In the following section we report the results of solving the IPD in the co-action framework both for the 2-player as well as the *N*-player scenarios. We conclude with a brief discussion of the implications of our results for the evolution of cooperation and related issues.

## The co-action solution concept

The conventional Nash solution of a game defines a set of strategy choices by agents such that no one gains by unilateral deviation, i.e., altering only her strategy while assuming that those of others remain fixed. However, as we have shown recently for a *single-stage* game^22^, the assumptions underlying the Nash framework are mutually inconsistent when the game situation is symmetric (i.e., exchanging the identities of the agents leaves the payoff structure invariant). Specifically, assuming that (a) each agent is aware that all agents are equally capable of analyzing the game situation, is inconsistent with the assumption that (b) agents can make unilateral deviations in their strategy - a necessary premise for obtaining a dominant strategy. Removing this inconsistency yields the co-action equilibrium^22^,^24^ where each agent is aware of the symmetric situation that all agents are in. Thus an agent will realize that, whatever strategy choice she is going to make, other agents, being in the same symmetric situation and being just as rational as her, will make too. Simply put, this is the only logical conclusion that can be arrived at by a rational agent under such conditions. Note that this does not imply that agents will necessarily choose the same action, e.g., if they are using mixed strategies as may happen in PD for a certain range of payoff values as discussed below.

**Figure 1:**
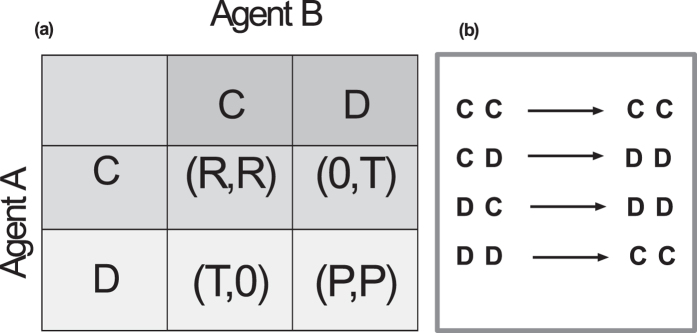
(**a**) A generic representation of the payoff matrix for a two-person PD (*T* > *R* > *P* > 0, with the sucker’s payoff *S* assumed to be zero for convenience). At every round, each agent can choose one of two possible actions, cooperate (C) or defect (D). For each pair of actions, the first entry in each payoff pair belongs to Agent A while the second belongs to Agent B. (**b**) Representation of the Pavlov strategy corresponding to the co-action solution of the two-agent IPD. The arrows connect the optimal actions of Agents A and B (in order) in the present round to information about their actions in the previous round.

We illustrate this distinction between the Nash and co-action frameworks in the single-stage PD representing a one-off strategic encounter between two agents who have the choice to either cooperate (*C*) or defect (*D*). In this game, if both agents choose *C*, each receives a reward payoff *R*, while if they both choose *D*, each is penalized with a punishment payoff *P* < *R*. If they choose different actions, then the defector receives the highest payoff *T* > *R* (the temptation to defect) while the cooperator gets the lowest (or sucker’s) payoff *S* < *P* [see the payoff matrix in Fig. 1(a)]. Thus, the payoffs are ordered as *T* > *R* > *P* > *S*, for which it is easy to see that mutual defection is the only Nash equilibrium. In contrast to the Nash solution, co-action leads to a “cooperative” outcome resulting from the agents maximizing their payoffs under the assumption that other agents will use the same strategy as them (although they may not necessarily choose the same action - *C* or *D* - if the strategy is a probabilistic one). In the single-stage PD, this amounts to determining that value of *p*, i.e., the probability that an agent will choose *C*, for which the expected payoff function for each of the agents 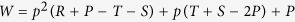 is maximum. Note that, here we have used the key concept of co-action, viz., that each agent will independently choose *C* with the same probability *p*. It is easy to see that this optimization problem has a unique solution corresponding to the agents always cooperating when *T* ≤2*R* and cooperating with a probability 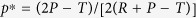 when *T* > 2*R*^22^.

The above argument does not take into account the possibility of previous interactions among the agents. In other words, there is no consideration of any memory of how the agents behaved in previous rounds. However, many strategic interactions that arise in biological, economic and social contexts are iterative in nature, where individuals can engage with each other repeatedly. If the agents are capable of recalling how their opponents acted in earlier interactions, this information can be used by them to formulate their current strategies. The phenomenon of direct reciprocity^11^ can be placed in this general context, providing a platform for addressing the problem of evolution of cooperation through the Iterated Prisoners Dilemma (IPD). In contrast to the single stage game described above, the IPD involves two players repeatedly playing the game. Just as for the single stage game, mutual defection is the only Nash equilibrium for a finitely repeated IPD, which can be easily shown by a backward induction argument. In an infinitely repeated IPD however, it is possible to have mutual cooperation as an equilibrium outcome, as indicated by folk theorems^25^. Computerized tournaments in which different programs are made to play IPD against each other have indeed shown the success of strategies that incorporate reciprocity, such as TFT and Pavlov, which can help maintain cooperation^1^. It would be a significant theoretical breakthrough if any of these reciprocal strategies can be shown to be the rational solution of IPD - even in a restricted context such as that of 1-step memory rules (i.e., those which take into account the history of only the previous round). As we show below, this can be achieved using the co-action solution concept. Note that direct reciprocity allows the knowledge gained from previous interactions to be used by agents to infer the existence of symmetry - even in the absence of any communication between them - which is the crucial ingredient for the co-action concept to apply. Thus, generalizing the co-action framework which had been originally proposed in the context of one-shot games to an iterative setting allows its application to a wide class of non-cooperative strategic interactions in nature where symmetry between the players need not be assumed a priori.

## Results

### IPD between two agents

The co-action solution for the case of two agents playing IPD can be derived as follows. Consider the payoff matrix for a single round of interaction between the agents as shown in Fig. 1(a). The value of the lowest payoff *S* is assumed to be zero without loss of generality. In addition, we consider the case 2*R* > *T* so as to rule out the possibility of a strategy in which agents take turns to alternately cooperate and defect. For the sake of clarity, we look at 1-step memory strategies where each agent has the knowledge of the choice made by all agents in the last round. Similar considerations will apply when extending the analysis to longer-memory strategies.

In the co-action equilibrium, the symmetry of the game situation as perceived by the agents governs their strategies. As the agents can recall their actions in the immediately preceding round of the game, if both had chosen the same action (i.e., *CC* or *DD*), this is recognized as establishing complete symmetry between the agents - in which case, they behave as in the single-stage PD co-action solution^22^. If, on the other hand, each had chosen a different action (i.e., *CD* or *DC*), then the agents realize that they are not in a symmetric situation and will resort to Nash-like reasoning.

To set out the argument in detail, we consider the four different possibilities that can arise during the course of the IPD, viz., (i) agent *A* cooperated while agent *B* defected (*CD*), (ii) both cooperated (*CC*), (iii) both defected (*DD*) and (iv) agent *A* defected while agent *B* cooperated (*DC*), in the last round. Thus, the state an agent is in at any given time could be any one of the following: 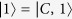, 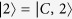, 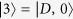 and 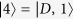. In this notation, the first entry denotes whether the agent cooperated (*C*) or defected (*D*) and the second entry denotes the total number of agents who cooperated in the previous round. If *p*_*i*_ denotes the probability with which an agent in state 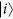 switches her action, we can express her expected payoffs *W*_*i*_ in the different states as:


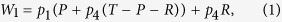



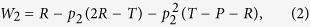



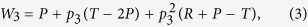



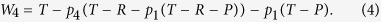


Note that the payoff *W*_2_ is a function of only *p*_2_ and *W*_3_ is a function of only *p*_3_, as, in the co-action framework, both the agents in these states (corresponding to *CC* and *DD*, respectively) are in a completely symmetric situation. Hence, the agents in state 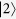 (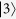) will each switch to defection (cooperation) with the same probability *p*_2_ (*p*_3_). It is easily seen that the values for *p*_2_ and *p*_3_ that maximize the respective payoff functions *W*_2_ and *W*_3_ are 0 and 1, respectively (corresponding to mutual cooperation).

For the states 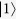 and 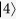 (corresponding to *CD* and *DC*, respectively), where the agents are not in a symmetric situation, agent in state 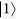 will try to maximize *W*_1_ by varying *p*_1_ for any given value of *p*_4_ while the agent in state 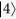 will seek to maximize *W*_4_ by varying *p*_4_ for any given value of *p*_1_. Using the same reasoning that is employed to obtain the Nash strategies, it is easy to see that the only mutually consistent choice for the optimal strategies of the two agents is 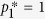 and 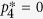 (corresponding to mutual defection). The optimal strategies for the agents in different states are summarized in Fig. 1(b). Hence, the agents will resort to co-action thinking whenever they find themselves in a symmetric situation (as in *CC* or *DD*) while they use Nash-like reasoning in other situations (as in *CD* or *DC*). In the latter case, they will arrive at a symmetric situation in the next round (as they choose *DD*), and thereafter will mutually cooperate.

An important observation about the co-action solution of the two-person IPD discussed above is that the optimal strategy [Fig. 1(b)] turns out to be the same as the Pavlov strategy for IPD proposed by Nowak and Sigmund^26^. This strategy has been shown to have certain advantages over the well-known tit-for-tat (TFT) strategy^1^ for playing IPD, viz., it can correct for occasional mistakes in implementation of strategies and can exploit unconditional cooperators^27^,^28^. More generally, Pavlov type of behavior has been widely observed in natural situations^29^, including experimental realizations of PD^30^. We emphasize that unlike in earlier studies where the Pavlov strategy is considered as an *ad-hoc* behavioral rule for agents, here we have demonstrated from first principles that such a strategy is the optimal solution for rational, selfish agents in the two-agent IPD.

### IPD between many agents

We now consider an IPD with *N*(>2) agents, each of whom play with all the others in every round. An individual chooses an action (either *C* or *D*) in each round which it employs against everyone else in that round, receiving payoffs for each pairwise interaction according to the matrix in Fig. 1(a). As in the two-agent case, we assume that *S* = 0 and 2*R* > *T*. In addition, we set the “punishment” payoff *P* to 0 for simplicity (alternatively, one can consider 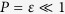
31). The total payoff received by an agent in any round is the sum of the individual payoffs from each of the (*N*−1) two-agent interactions. This ensures that all the agents receive a lower payoff if everyone defects than if they all cooperate, and if any agent switches from *D* to *C*, the average payoff of the agents increases.

The above situation describes an instance of *public goods dilemmas* where individual contributions towards a public good increases the collective benefit although the cost borne by an individual for this contribution exceeds the benefit she derives from it^10^,^32^. While the general problem of public goods has been considered under various guises in the literature^33^, in the simple quantitative setting involving a well-mixed population as described above, it is easy to see that a single round of interaction in a *N*-person public goods game is equivalent to *N*−1 pairwise PD interactions^34^. This does not imply that the situation described by the public goods dilemma simply corresponds to a quantitative increase in the number of agents of the PD game, but rather involves a profound change in the nature of the interactions^35^. Agents can react only to the combined effect of the actions of all other agents and not to the individual strategies of specific agents. The anonymity provided to individuals in the multi-player setting means that they are more likely to defect (i.e., act as free-riders) without much fear of retaliation by others^23^.

The state that an agent is in at any given time can be represented by either 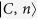 or 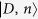  according to whether she cooperated (*C*) or defected (*D*) in the previous round, with *n* denoting the total number of agents who cooperated in the previous round. In the co-action framework, the set of agents who played *C* in a particular round realizes that all of them who chose *C* are in a symmetric situation. Similarly, the set of agents who played *D* are aware of the symmetry among them. Thus, within each group, all agents will use identical strategies for the next round. For simplicity, we consider only pure strategies where agents choose either *C* or *D* with probability 1^23^,^36^,^37^,^38^.

Let us first consider the two extreme cases corresponding to either everyone cooperating or everyone defecting in the previous round. If all the agents had cooperated, they would realize that all of them would use identical strategies. The expected payoff of any agent is simply an integral multiple of *W*_2_ (see Eq. 2), the corresponding payoff in the two-agent case studied earlier. Thus, on optimizing payoff, all agents choose *C* in the next round. By similar arguments, if all agents had chosen to defect in the previous round, they would choose *C* in the next round.

**Figure 2:**
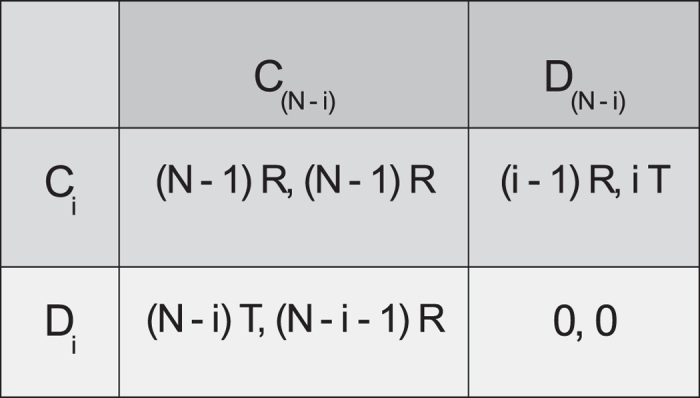
Representation of possible payoffs in an *N*-player IPD under the co-action framework, when *i* agents cooperated and *N*−*i* agents defected in the previous round.

When some of the *N* agents cooperate and the others defect, we can treat the situation as identical to a two-player scenario, with the Nash equilibrium being the optimal strategy. Note however that each “player” is now a group of agents and the corresponding Nash solution is distinct from the one corresponding to everyone defecting as is obtained in a conventional 2-person PD. The expected payoffs of the two sets of agents can be conveniently represented by a two-player payoff matrix as shown in Fig. 2. Here the row corresponds to the set of *i* agents (where 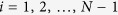) who cooperated in the last round, while the column corresponds to the set of 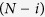 agents who defected. In the next round, the row “player” can either choose to continue cooperating (*C*_*i*_) or switch to defection (*D*_*i*_). Similarly, the column “player” can switch to cooperation in the next round (*C*_*N−i*_) or continue to defect (*D*_*N−i*_). Thus, starting with any combination of cooperating and defecting agents, we can obtain the optimal strategies for the two sets of agents which depend on the ratio of the payoffs *T*/*R* for a given *i*.

The co-action solution for the four possible situations that can arise in terms of the relative magnitudes of the payoffs for the two sets of agents are:

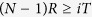
 and 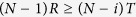: From Fig. 2, it is clear that cooperation is the optimal choice for both the sets of agents as neither will benefit by deviating from this strategy.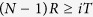 and 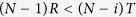: It is easy to see that cooperation is the optimal choice for the column “player” independent of the action of the row “player”, and using this information, one observes that the optimal choice for the row “player” would be to defect. Thus, the set of agents who cooperated in the previous round will switch to defection, while the set which defected will switch to cooperation.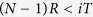 and 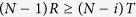: Again it is easy to see that cooperation is the optimal choice for the row “player” independent of the action of the column “player”, and using this information, one observes that the optimal choice for the column “player” would be to defect. The agents will therefore continue with the same actions as in the previous round.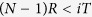 and 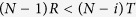: This situation arises only when *i* = *N*/2 (and hence only for even values of *N*), i.e., when there are equal number of cooperators and defectors. For this case, there are two possibilities for the optimal action, one where the “players” continue with the same action as in the previous round, and, the other where each of them switches to the opposite action. Note that the level of cooperation does not change in either of the cases.

For illustrative purpose, we now discuss in detail the co-action solution of the *N*-person IPD for the cases when *N* = 3, 4 and 5. In each of these cases, we shall denote the distinct states that are possible for the system to be in as *S*_*j*_ where 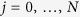 is the number of cooperators in that state. For *N* = 3 agents, it is easy to see by referring to the general co-action solution given above that the optimal strategies will result in the following evolution between the distinct states of the system: 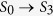, 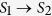, 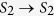 and 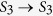. Thus, if all three agents had chosen the same action (*C* or *D*) in the previous round, all of them cooperate in the next round (*S*_3_). In all the other cases, the system converges to the state *S*_2_ corresponding to two cooperators and one defector. This result clearly distinguishes the co-action approach from the conventional Nash solution, which would have corresponded to all three defecting. A notable feature of the co-action solution is the stable coexistence of cooperators and defectors (as in state *S*_2_).

For the case when *N* = 4, as before by referring to the general co-action solution above, we see that the optimal strategies will result in the following evolution between the distinct states of the system: 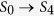, 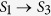, 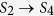 (if 3*R* ≥ 2*T*) or 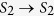 (otherwise), 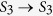 and 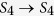. We can see that for *N* = 4 (unlike for *N* = 2 and 3) the solution begins to depend on the ratio of *T* to *R*, which is also true for all higher values of N.

As a final example, we consider the case when *N* = 5. Here the optimal strategies depend on whether the magnitude of the payoff values satisfy 4*R* > 3*T*. If this is true, it will result in the following evolution between the distinct states of the system: 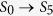, 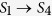, 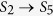, 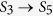, 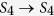 and 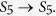 On the other hand, if 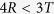, the following evolution will be observed: 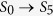, 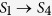, 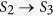, 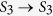, 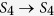 and 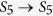.

Thus, we can draw the following general conclusions: (a) the state in which everybody cooperates (i.e., *i* = *N*) is a stable state, (b) a state in which all but one agent cooperate (*i* = *N*−1) is also a stable state, (c) states where the defectors are in a minority are stable if 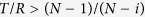 and (d) when the cooperators are in a minority, in the next iteration all agents will cooperate if 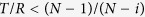, otherwise they will switch their respective choices (from C to D and vice versa). In the special case when *N* is even with exactly half of the agents cooperating and 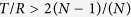, multiple equilibria are possible. The most important point to note from the above results is that cooperators can coexist with defectors, and moreover, always form a majority, in the co-action solution of the *N*-player IPD.

## Discussion

In contrast to the conventional wisdom that defection should be the preferred strategy of selfish agents, human subjects playing PD in either single-stage or multiple round experiments do achieve some measure of cooperation (e.g., see ref. 39). Understanding how such cooperation arises can be investigated in the context of repeated interactions between agents. In this case, agents can “remember” their past actions and the resulting outcomes, and they can use this information to govern their future decisions - a phenomenon referred to as direct reciprocity^12^. Apart from this, several other mechanisms for the emergence of cooperation through natural selection have been proposed^40^, such as, kin selection^41^, indirect reciprocity^42^, network reciprocity^31^,^43^ and group selection^44^. Even within the conventional game theoretic framework, there have been formal attempts at modifying IPD so as to make cooperation viable, involving concepts such as ε-equilibria^45^, incomplete information^46^, bounded rationality^47^, absence of common knowledge about the rationality of players^48^ and the number of iterations^49^, etc. In recent times there has been an increased focus on the evolution of cooperation in spatially extended situations where agents interact only with their neighbors defined by an underlying connection topology^31^,^50^,^51^,^52^,^53^.

In this paper we have addressed the question of whether a strategy incorporating direct reciprocity that allows for cooperation to be maintained can emerge as a rational solution of IPD. The novel perspective that we bring to bear involves recognizing the symmetry between agents - a crucial ingredient for the co-action framework to apply. In an iterative setting, agents become aware of their symmetry with other agents through the knowledge of their actions in previous encounters. The most important result of our study is that cooperators and defectors coexist in the co-action solution of the *N*-player IPD; moreover, the majority of agents cooperate. This is remarkable in view of the conventional wisdom that cooperation is extremely difficult to achieve in a group of *selfish rational* agents^1^. For the case of two players, the co-action solution of IPD corresponds to the well-known Pavlov strategy that has been attested in animal behavior and social interactions^26^. To the best of our knowledge, the approach we present here is the only one that provides a rational game-theoretic basis for such a strategy, as opposed to proposing it as a *ad hoc* behavioral rule. It also allows the generalization of Pavlov to the situation of multiple (*N* > 2) agents.

An important consideration in studies of IPD is the role of noise that can arise from the incorrect implementation of intended action by agents^54^,^55^. Such noise may also be due to the misinterpretation of actions of other agents^56^. For example, the TFT strategy in IPD is vulnerable to such noise as it cannot correct for occasional mistakes by agents. While for the case of two players it is known that the Pavlov strategy (which is the co-action solution for *N* = 2) is stable in the presence of noise^57^, it is easy to see that even in the case of *N* > 2 agents, the co-action solution is not significantly affected by intermittent errors on the part of the agents.

The iterative game situation considered here corresponds to 1-step memory where the agents only retain information about the action of other agents in the immediately preceding round. The co-action concept can be easily extended to the more general situation of agents with longer memories, once the key question of how the symmetry among agents is to be defined in such a setting is addressed. One possibility is that all agents who have cooperated an equal number of times in the past are considered to be in a symmetric situation. They will therefore adopt the co-action strategy in the next round. For two agents with finite memory, this will eventually lead to both of them cooperating. If there are more than two agents, the co-action principle suggests that those who display similar propensities to cooperate - i.e., they have cooperated an equal number of times in the past - will form a group defined by complete symmetry among the agents comprising it. Thus, the entire set of *N* agents can be divided into a number of such “symmetry groups”. This defines a novel class of strategic interactions where the “players” are the different symmetry groups (each consisting of one or more agents) playing according to strategies given by the Nash equilibrium. It is important to point out that this will not result in all agents resorting to defection as expected in the conventional Nash framework. Potentially, this new class of games can be used to analyze multi-agent strategic interactions in many different contexts.

It is intriguing to consider the implications of the co-action strategy for the behavior of individuals in real-world social interactions. As we show here, the stable solutions are those where a majority of agents cooperate, suggesting that the presence of a few defectors will not necessarily result in the breakdown of cooperation in a society. This is because rational agents who perceive each other to be similar, will not be deterred from cooperating as long as they receive enough mutual support - in the form of acts of cooperation - from similar agents. The co-action framework, therefore, implies that significant levels of cooperation will be seen in interactions among rational individuals in IPD-like situations, in contrast to conventional wisdom. There have been a large number of experiments carried out with human subjects playing IPD (both the 2-person as well as the multiple-player version, viz., the repeated public goods game). Surveying the results reported in many experiments over several decades reveal that, both for the two-person IPD^39^, as well as, the repeated public goods game^58^,^59^, the majority of experimental subjects do not behave in the manner predicted by conventional game theory. As shown in this paper, the co-action paradigm provides a mechanism for a rational explanation of experiments on IPD that do not show complete absence of cooperation. It can also perhaps help in understanding cooperative behavior seen among non-human animals who do not share kinship^20^, a phenomenon that has been experimentally investigated in an IPD framework, e.g., in birds^18^.

The setting in which we have discussed the problem of evolution of cooperation above corresponds to the idealized situation of fully rational agents interacting with each other. While the rationality assumption is used widely in situations involving human actors, one can ask how the co-action paradigm may apply to other animals or even colonies of unicellular organisms where the emergence of cooperative behavior is observed^4^,^5^,^6^,^7^,^8^. As symmetry is the crucial ingredient for the co-action framework to be valid, it is not unreasonable to apply it for interactions among members of a homogeneous population who share a common identity. This homogeneity could be in terms of, for example, the genetic composition, physiognomy or even acquired traits. Depending on the specific context in which the evolution of cooperation is being considered, one or more of these identities could be relevant for the co-action framework to apply. For instance, tag-based cooperation among “similar” individuals^60^ could arise naturally under this framework.

To conclude, we have shown that the co-action paradigm provides a new perspective to the evolution of cooperation. The co-action concept has been earlier shown to resolve social dilemmas in single-stage symmetric games. Here we show how the idea of co-action applies to the more general setting of iterative game situations. Information about previous interactions allows agents to infer symmetry (or its absence) among themselves, allowing cooperation to emerge even when agents had initially chosen to defect. The co-action framework also provides a rational basis for the Pavlov strategy that has been proposed for the two-person IPD, and generalizes such a strategy to the case of several agents. In general, we observe that cooperators and defectors can coexist in the *N* player Iterated Prisoners Dilemma game, with the cooperators constituting the majority. This is a surprising feature given the conventional expectation that selfish, rational agents will always defect.

## Additional Information

**How to cite this article**: Sasidevan, V. and Sinha, S. Co-action provides rational basis for the evolutionary success of Pavlovian strategies. *Sci. Rep.* **6**, 30831; doi: 10.1038/srep30831 (2016).
